# USP13 deubiquitinates and stabilizes cyclin D1 to promote gastric cancer cell cycle progression and cell proliferation

**DOI:** 10.1038/s41388-023-02739-x

**Published:** 2023-06-13

**Authors:** Cunying Ma, Dandan Wang, Zhuangfei Tian, Wenrong Gao, Yichen Zang, Lilin Qian, Xia Xu, Jihui Jia, Zhifang Liu

**Affiliations:** 1 Department of Biochemistry and Molecular Biology, Key Laboratory for Experimental Teratology of Chinese Ministry of Education, School of Basic Medical Sciences, Cheeloo College of Medicine, Shandong University, Jinan, PR China; 2 Department of Microbiology, Key Laboratory for Experimental Teratology of Chinese Ministry of Education, School of Basic Medical Sciences, Cheeloo College of Medicine, Shandong University, Jinan, PR China

**Keywords:** Gastric cancer, Ubiquitylation

## Abstract

The reversible post-translational modifications of protein ubiquitination and deubiquitination play a crucial regulatory role in cellular homeostasis. Deubiquitinases (DUBs) are responsible for the removal of ubiquitin from the protein substrates. The dysregulation of the DUBs may give rise to the occurrence and development of tumors. In this study, we investigated the gastric cancer (GC) data from the TCGA and GEO databases and found that ubiquitin-specific protease USP13 was significantly up-regulated in GC samples. The higher expression of USP13 was associated with the worse prognosis and shorter overall survival (OS) of GC patients. Enforced expression of USP13 in GC cells promoted the cell cycle progression and cell proliferation in an enzymatically dependent manner. Conversely, suppression of USP13 led to GC cell cycle arrest in G1 phase and the inhibition of cell proliferation. Nude mouse experiments indicated that depletion of USP13 in GC cells dramatically suppressed tumor growth in vivo. Mechanistically, USP13 physically bound to the N-terminal domain of cyclin D1 and removed its K48- but not K63-linked polyubiquitination chain, thereby stabilizing and increasing cyclin D1. Furthermore, re-expression of cyclin D1 partially reversed the cell cycle arrest and cell proliferation inhibition induced by USP13 depletion in GC cells. Additionally, USP13 protein abundance was positively correlated with the protein level of cyclin D1 in human GC tissues. Taken together, our data demonstrate that USP13 deubiquitinates and stabilizes cyclin D1, thereby promoting cell cycle progression and cell proliferation in GC. These findings suggest that USP13 might be a promising therapeutic target for the treatment of GC.

## Introduction

Gastric cancer (GC) ranks fifth in terms of global tumor incidence and fourth in tumor related mortality in the world [1]. There are over one million new cases in 2020 and an estimated 769,000 deaths according to the epidemiological reports [1]. Despite significant improvements in the study, diagnosis and treatment of the tumor, most of advanced and metastatic GC patients confront the poor prognosis and low 5-year survival rate [2]. Thus, it is urgent to find effective diagnostic biomarkers and therapeutic targets for the tumor.

Ubiquitination modification of proteins is one of the most important protein post-translational modifications and serves to mediate protein degradation or regulate protein function [3]. The ubiquitination process of proteins is catalyzed by ubiquitin activating enzyme (E1), ubiquitin coupling enzyme (E2) and ubiquitin-protein ligase (E3), which can be reversed by deubiquitinases (DUBs) [4]. DUBs are responsible for removing the ubiquitin chain(s) from substrate proteins and thus regulate the stability of the substrates [5]. An enormous number of studies have demonstrated that disrupted regulation of protein deubiquitination contributes to the occurrence and development of multiple diseases, especially cancers [6]. Due to the greater numbers, diversity and well-defined catalytic regions, DUBs have great potential as drug targets [6, 7].

As a DUB, ubiquitin-specific protease 13 (USP13) plays important roles in various physiological and pathological processes, such as cancer cell stemness [8, 9], epithelial-mesenchymal transition and metastasis [10], mitochondrial energy metabolism [11], autophagy [12, 13], DNA damage response [14] and cell cycle [15] by regulating the deubiquitination of a variety of key substrate proteins, including c-Myc [8], FASN [9], Snail1 [10], ATP citrate lyase, oxoglutarate dehydrogenase [11], VPS34 [12], PIK3C3 [13], RAP80 [14] and Aurora B [15]. The dysregulation of USP13 expression is associated with the tumorigenesis and progression of tumor. However, the roles of USP13 in different tumor types are controversial. It was reported that USP13 functions as a tumor suppressor in bladder cancer [16], breast cancer [17] and oral squamous cell carcinoma [18]. In these tumors, USP13 interacts with and deubiquitinates the tumor suppressor PTEN protein, thereby stabilizing and increasing the expression of PTEN [16–18]. In contrast, USP13 is considered as an oncogene in other tumors, such as in cervical cancer [19], lung cancer, ovarian cancer [20], glioblastoma [8], melanoma [21] and kidney cancer [22]. In these tumors, USP13 deubiquitinates and stabilizes some oncogenes, including MCL1 [19, 20], Myc [8], MITF [21] and ZHX2 [22]. Although it has been reported that USP13 acts as an oncogene in GC [10], its biological function and potential target substrates have not been fully explored.

In this study, we investigated the expression and biological function of USP13 in GC and identified its novel substrate target. We confirmed the up-regulation of USP13 in human GC tissues. The higher expression of USP13 was associated with the poorer prognosis of the GC patients. Enforced expression of USP13 promoted the cell cycle progression and cell proliferation by enhancing the protein stability of cell cycle protein cyclin D1. USP13 interacted with and deubiquitinated cyclin D1. USP13 was positively associated with cyclin D1 in human GC specimens. Taken together, our work identified a new substrate for USP13 in regulating cell cycle progression and revealed that USP13 may represent a promising diagnostic marker and a potential therapeutic target for GC.

## Results

### USP13 is highly expressed in GC tissues

**Fig. 1:**
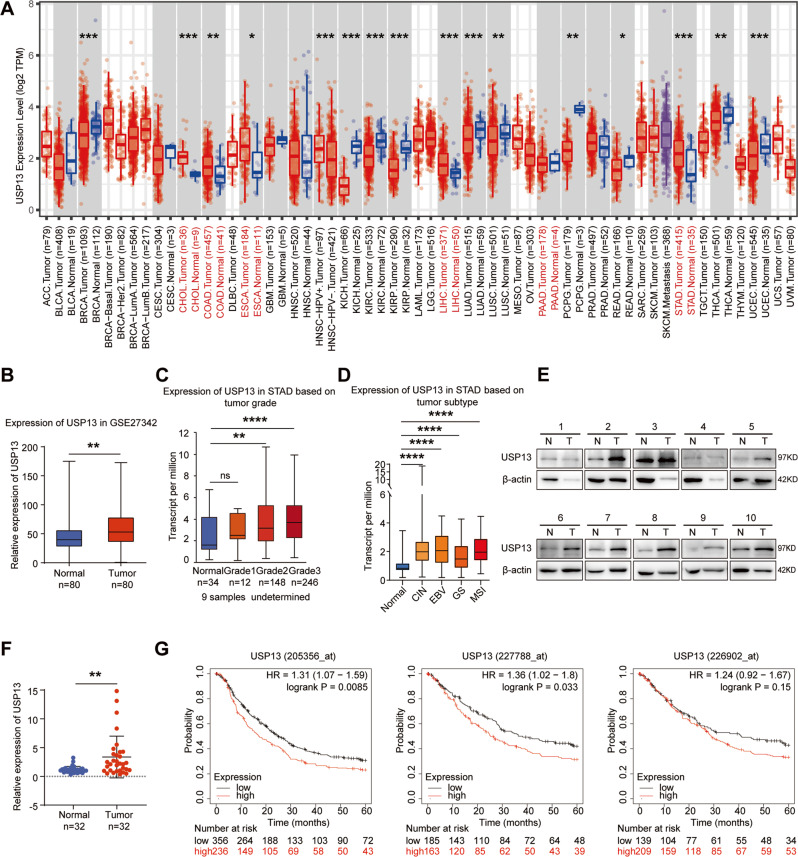
The expression level of USP13 is increased in GC tissues, and the higher expression of USP13 is correlated with shorter overall survival (OS) of GC patients. **A** USP13 mRNA expression level in different tumor tissues and non-tumor tissues from TCGA database was analyzed with TIMER 2.0 (http://timer.cistrome.org/) [42]. The red dots represent tumor tissues, while the blue dots represent normal tissues. ACC Adrenocortical Carcinoma, BLCA Bladder Urothelial Carcinoma, BRCA Breast Invasive Carcinoma, CESC Cervical and Endocervical Cancer, CHOL Cholangiocarcinoma, COAD Colon Adenocarcinoma, DLBC Diffuse Large B-cell Lymphoma, ESCA Esophageal Carcinoma, GBM Glioblastoma Multiforme, HNSC Head and Neck Cancer, KICH Kidney Chromophobe, KIRC Kidney Renal Clear Cell Carcinoma, KIRP Kidney Renal Papillary Cell Carcinoma, LAML Acute Myeloid Leukemia, LGG Lower Grade Glioma, LIHC Liver Hepatocellular Carcinoma, LUAD Lung Adenocarcinoma, LUSC Lung Squamous Cell Carcinoma, MESO Mesothelioma, OV Ovarian Serous Cystadenocarcinoma, PAAD Pancreatic Adenocarcinoma, PCPG Pheochromocytoma and Paraganglioma, PRAD Prostate Adenocarcinoma, READ Rectum Adenocarcinoma, SARC Sarcoma, SKCM Skin Cutaneous Melanoma, STAD Stomach Adenocarcinoma, TGCT Testicular Germ Cell Tumors, THCA Thyrold Carcinoma, THYM Thymoma, UCEC Endometrioid Carcinoma, UCS Uterine Carcinosarcoma, UVM Uyeal Melanoma. **B** Analysis of mRNA expression level of USP13 in GC and noncancerous tissues based on GEO (GSE27342) database. **C** Analysis of the mRNA expression of USP13 in GC tissues based on the tumor grades according the data from TCGA database. **D** Analysis of the mRNA expression of USP13 in normal and different GC subtypes (CIN chromosomal instability, EBV Epstein–Barr virus, GS genomically stable, MSI microsatellite instability) according to the data from TCGA database and GTEx database. **E** Western blot was used to detect the protein expression of USP13 in 32 pairs of GC tissues and the adjacent nontumor tissues of GC patients (N non-tumor tissue, T tumor tissue). The representative results were showed. **F** The protein expression levels of USP13 in the GC tissues and the corresponding nontumor tissues were determined with the ImageJ software and normalized to the expression of β-actin. The data were statistically analyzed using GraphPad Prism v8.0 2 software with a paired Student’s *t* test. **G** Kaplan–Meier Plotter database was used to analyze the overall survival (OS) of GC patients from the pooled analysis excluding GSE62254. The results were from 3 different Affymetrix probes. *p < 0.05; **p < 0.01; ***p < 0.001; ****p < 0.0001.

First, we used the Cancer Genomics Atlas (TCGA) database to investigate the level of USP13 transcript in different tumor tissues and found that USP13 was up-regulated in most of the digestive system neoplasms, including GC (Fig. 1A). The data from GEO dataset (GSE27342) also exhibited the up-regulation of USP13 in GC tissues (Fig. 1B). Then we analyzed the expression of USP13 in different GC grades and subtypes. The results indicated that the expression of USP13 was positively associated with the tumor grades (Fig. 1C). And compared to normal, the expression of USP13 was significantly increased in four different GC subtypes, with the highest average expression value in the EBV (epstein–barr virus) subtype and the lowest expression in the GS (genomically stable) subtype (Fig. 1D). We further used western blot to determine the protein level of USP13 in 32 pairs of GC and adjacent noncancerous tissues and found that the protein level of USP13 was elevated in 65.6% (21/32) of the GC tissues (Fig. 1E). We used ImageJ software to quantify the protein bands and statistically analyzed the data with paired *t-test*. The results showed that the protein level of USP13 was significantly increased in GC tissues (*p* < 0.01) (Fig. 1F). Additionally, we used Kaplan–Meier Plotter database to analyze the correlation between USP13 exprsssion and the overall survival (OS) of GC patients. Since the dataset GSE62254 in Kaplan–Meier Plotter database contains markedly different characteristics from all other datasets, we excluded GSE62254 from pooled analysis and analyzed GSE62254 separately. The results from three different Affymetrix probes (205356_at, 227788_at, 226902_at) showed similar trends that higher expression of USP13 in GC tissues resulted in shorter overall survival (OS) of GC patients both in the pooled analysis excluding GSE62254 (Fig. 1G) and in the separate analysis of GSE62254 (Supplementary Fig. S1).

### USP13 knockdown inhibits cell cycle progression and cell proliferation in GC

**Fig. 2:**
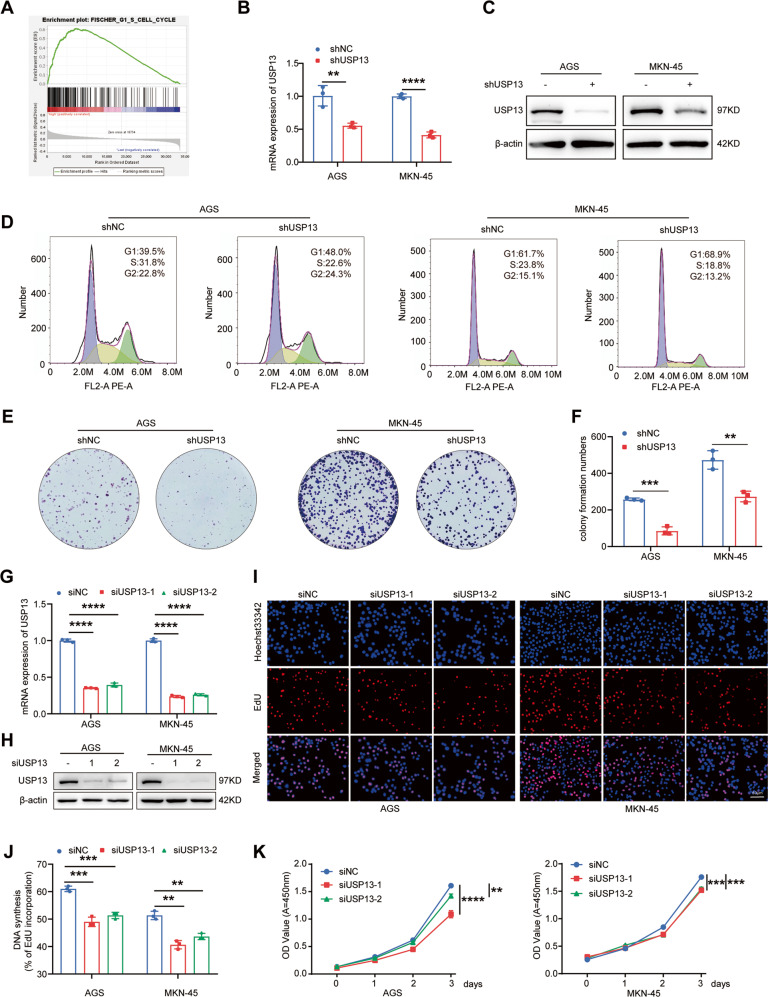
Knockdown of USP13 induces GC cell cycle arrest in G1 phase and inhibits cell proliferation and colony formation ability. **A** Enrichment plots of gene expression signatures for cell cycle according to USP13 mRNA expression in a published cohort (http://www.cbioportal.org/, TCGA, Nature 2014). The barcode plot indexes the position of the genes in each gene set. The number of patient samples is 375. Red and blue colors indicate high and low level of USP13, respectively. **B** qRT-PCR was used to determine the mRNA level of USP13 in GC cells infected with negative control lentivirus (shNC) or harboring USP13 shRNA lentivirus (shUSP13). **C** Western blot was used to detect the protein expression of USP13 in GC cells infected with shNC or shUSP13 lentivirus. **D** Flow cytometry was used to analyze the cell cycle distribution in treated GC cells. Representative results were shown. **E** Colony formation ability was determined in treated GC cells. Representative results were shown. **F** Statistical analysis of the colony formation number in treated GC cells. The data are the means ± SD from three independent experiments. **G** qRT-PCR was used to determine the mRNA level of USP13 in GC cells transfected with negative control siRNA (siNC) or USP13 siRNA (siUSP13). **H** Western blot was used to detect the protein expression of USP13 in GC cells transfected with siNC or siUSP13. **I** EdU assay was used to determine the cell proliferation ability in transfected GC cells. The representative results were showed. **J** Statistical analysis of the EdU-positive cell ratio in GC cells. The data are expressed as the means ± SD from three independent experiments. **K** CCK-8 assay was used to detect cell proliferation ability. The data are the means ± SD from three independent experiments. **p < 0.01; ***p < 0.001; ****p < 0.0001.

The gene set enrichment analysis (GSEA) in a published GC cohort showed that USP13 was significantly associated with cell cycle (Fig. 2A). Therefore, we investigated the effect of USP13 on GC cell cycle progression and cell proliferation. We used lentivirus harboring negative control shRNA or USP13 shRNA to infect AGS and MKN-45 cells. qRT-PCR and western blot results confirmed that infection with lentivirus harboring USP13 shRNA obviously decreased the expression level of USP13 in GC cells (Fig. 2B, C). Then we used flow cytometry to detect the cell cycle distribution and found that depletion of USP13 led to the cell cycle arrest at G1 phase (Fig. 2D). The colony formation assay showed that depletion of USP13 obviously decreased the colony formation ability of GC cells (Fig. 2E, F). To further validate the effect of USP13 on GC cells, we designed two small inference RNAs (siRNAs) to specifically target USP13 and transfected them into the GC cells. The efficient knockdown of USP13 in the transfected GC cells were validated by qRT-PCR and western blot (Fig. 2G, H and Supplementary Fig. S2A, B). EdU and CCK-8 assays were used to determine the cell proliferation ability. The results demonstrated that USP13 knockdown significantly suppressed GC cell proliferation and colony formation ability (Fig. 2I–K and Supplementary Fig. S2C–F).

### USP13 overexpression promotes cell cycle progression and cell proliferation in a DUB enzyme activity dependent manner

To further investigate whether USP13 overexpression has the opposite effect, we transfected USP13 overexpression vector into the GC cells (Supplementary Fig. S3A,B). The cell cycle distribution was determined with flow cytometry, and the cell proliferation ability was detected with EdU and CCK-8 assays. The results showed that overexpression of USP13 led to a decrease of the cell number in G1 phase (Supplementary Fig. S3C) and an increase of the cell proliferation ability in GC cells (Supplementary Fig. S3D–F).

**Fig. 3:**
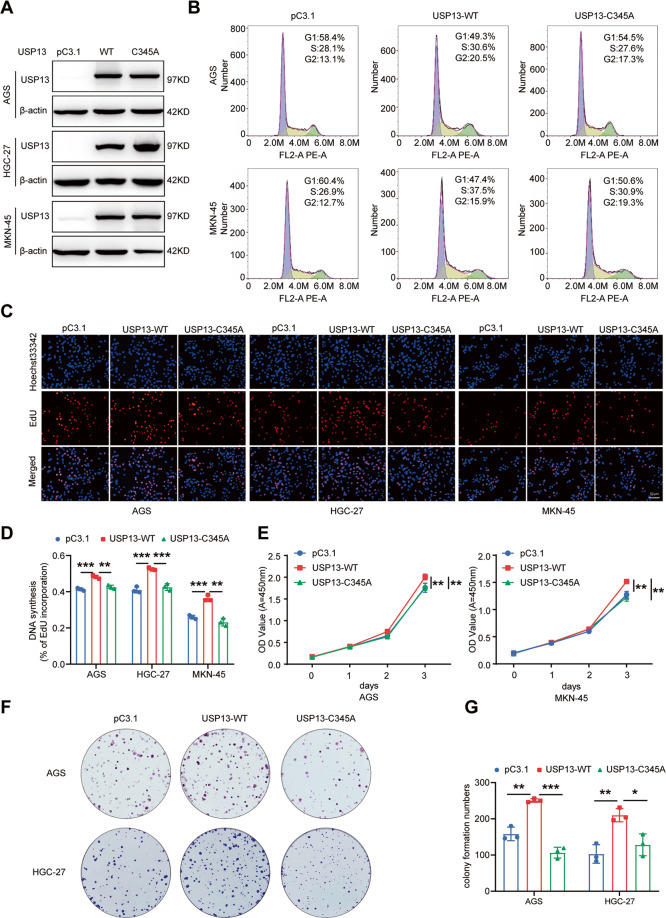
Enforced expression of wild-type USP13, but not the USP13 mutant C345A, promotes cell cycle progression and cell proliferation ability in GC cells. **A** Western blot was used to analyze the expression of USP13 in GC cells transfected with wild-type USP13 (WT) or catalytically inactive USP13 mutant C345A (C345A). **B** Cell cycle distribution in GC cells with different transfections was determined using flow cytometry. Representative results were shown. **C** The cell proliferation ability in transfected GC cells was detected with EdU assay. The representative results were showed. **D** Statistical analysis of the EdU-positive cell ratio in GC cells. The data are the means ± SD from three independent experiments. **E** Cell proliferation ability was analyzed with CCK-8 assay in different transfected GC cells. The data are the means ± SD from three independent experiments. **F** Colony formation ability was determined in different transfected GC cells. The representative results were showed. **G** Statistical analysis of the colony formation number in transfected GC cells. The data are the means ± SD from three independent experiments. *p < 0.05; **p < 0.01; ***p < 0.001.

We then asked whether the DUB activity of USP13 is essential for its biological function. We transfected the WT-USP13 vector and catalytically inactive mutant USP13-C345A into the GC cells, respectively. The transfection efficiency was verified (Fig. 3A). The cell cycle distribution, cell proliferation ability and colony formation ability were then determined. Our results showed that overexpression of WT-USP13 vector dramatically promoted the cell cycle progression (Fig. 3B), cell proliferation (Fig. 3C–E) and colony formation ability (Fig. 3F, G), while overexpression of USP13-C345A almost abrogated the cell proliferation and colony formation ability mediated by WT-USP13 vector transfection. These results revealed the DUB activity of USP13 is essential for its function.

### USP13 increases the stability of cyclin D1 in GC cells

**Fig. 4:**
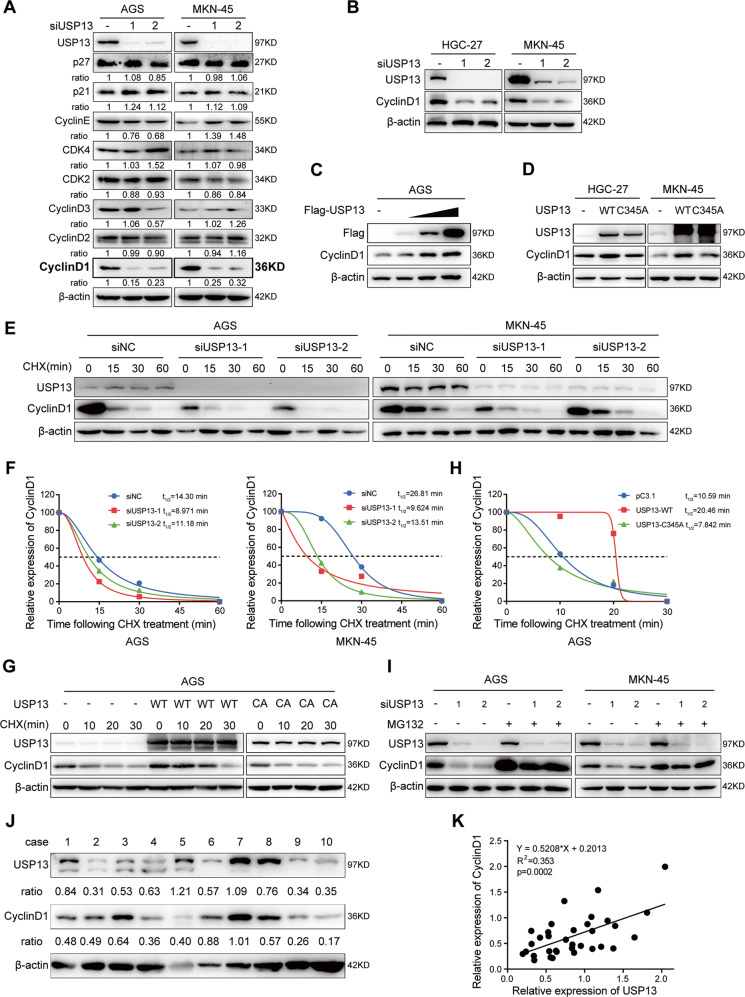
USP13 increases cyclin D1 expression and maintains its protein stability. **A** Western blot was used to detect the indicated gene expression in GC cells transfected with negative control siRNA or USP13 siRNAs (siUSP13). Cyclin D1 protein was detected using western blot in GC cells transfected with USP13 siRNAs (**B**) or increased concentration gradient of Flag-USP13 (**C**). **D** Cyclin D1 protein was detected using western blot in GC cells transfected with wild-type USP13 (WT) or mutant USP13-C345A (C345A). **E** Endogenous cyclin D1 degradation ratio was detected with western blot in GC cells transfected with negative control siRNA or USP13 siRNA and then treated with cycloheximide (CHX) for the indicated time intervals. **F** The quantification and statistical analysis of cyclin D1 protein levels in **E**. **G** Endogenous cyclin D1 degradation ratio was detected with western blot in GC cells transfected with wild-type USP13 or USP13-C345A mutant and then treated with CHX for the indicated time intervals. **H** The quantification and statistical analysis of cyclin D1 protein levels in **G**. **I** Endogenous cyclin D1 degradation was detected with western blot in GC cells transfected with negative control siRNA or USP13 siRNA and then treated with proteasome inhibitors MG132 (10 μM) for 6 h. **J** Western blot was used to detect the protein levels of USP13 and cyclin D1 in GC samples. The band density of USP13 and cyclin D1 was quantified using ImageJ software and normalized to the corresponding β-actin. Representative data were shown. **K** The correlation analysis of USP13 and cyclin D1 protein level in 34 GC samples.

Having obtained the biological roles of USP13 in GC, we next tried to investigate the potential regulatory mechanism. Since the function of USP13 is associated with the cell cycle progression, we investigated the impact of USP13 on cyclin D1 and a series of other cell cycle-related proteins, including cyclin D2, cyclin D3, cyclin E, CDK2, CDK4, p21 and p27. As shown in Fig. 4A, B, USP13 siRNAs remarkably decreased the cyclin D1 protein level, but had no obvious effect on other cell cycle related proteins. In contrast, enforced expression of USP13 increased the protein level of cyclin D1 in a dose-dependent manner (Fig. 4C), while the mutated USP13-C345A with lost enzymatic activity had no obvious effect on cyclin D1 expression (Fig. 4D). qRT-PCR results showed that USP13 knockdown or overexpression did not change cyclin D1 mRNA level in GC cells (Supplementary Fig. S4A, B). To further confirm that USP13 regulates endogenous cyclin D1 stability, we transfected the GC cells with USP13 siRNA or expression vector and then treated the cells with the protein synthesis inhibitor cycloheximide (CHX) at different durations. Our results showed that USP13 knockdown decreased the half-life of cyclin D1 and facilitated the degradation of cyclin D1 in GC cells (Fig. 4E, F), whereas enforced expression of WT USP13, but not the USP13-C345A mutant, prolonged the half-life of cyclin D1 and delayed its degradation (Fig. 4G, H). To further explore whether proteasome degradation pathway is involved in USP13-mediated stability of cyclin D1, we treated the transfected cells with proteasome inhibitor MG132 and found that MG132 alleviated USP13 siRNA-induced cyclin D1 downregulation (Fig. 4I). Taken together, these results indicated that USP13 stabilized cyclin D1 by suppressing its proteasome degradation. Additionally, we determined the protein abundance of USP13 and cyclin D1 in 34 GC samples and found that USP13 was positively correlated with cyclin D1 in these GC tissues (Fig. 4J, K).

### USP13 directly interacts with cyclin D1 in GC cells

**Fig. 5:**
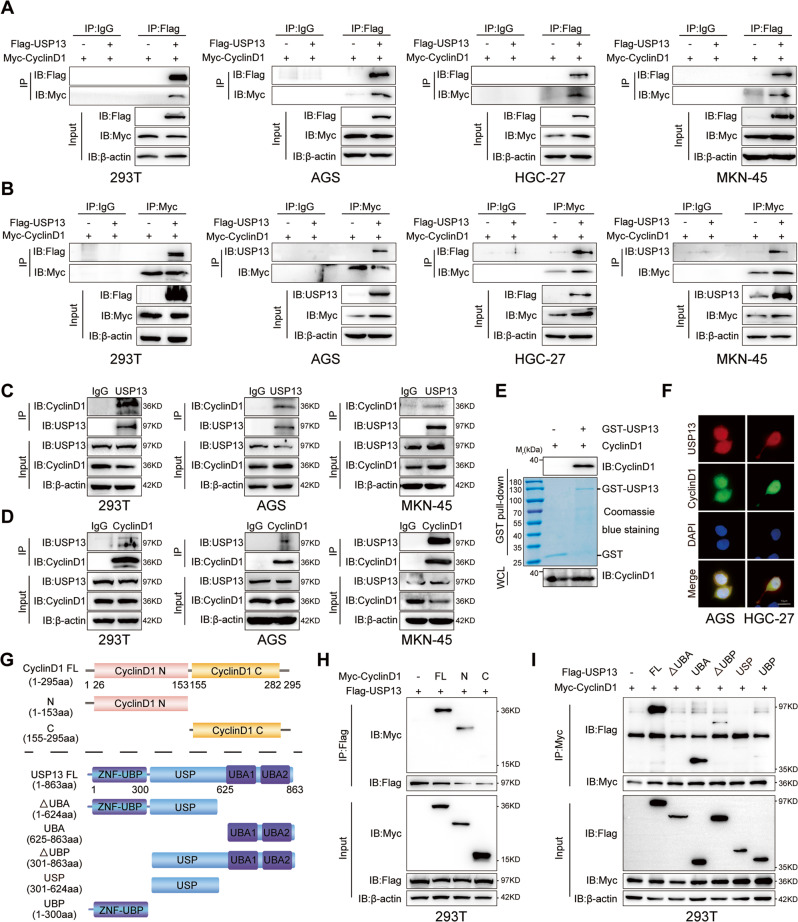
USP13 directly interacts with cyclin D1 in GC cells. The exogenous interaction between USP13 and cyclin D1 was detected with reciprocal immunoprecipitation analysis in HEK293T cells and GC cells transfected with empty vector or Flag-tagged USP13 in the presence of Myc-cyclin D1 and then subjected to immunoprecipitation with anti-Flag antibody to probe Myc-cyclin D1 (**A**) or anti-Myc antibody to probe Flag-USP13 (**B**). The endogenous interaction between USP13 and cyclin D1 was detected with reciprocal immunoprecipitation analysis in HEK293T cells and GC cells with anti-USP13 to probe cyclin D1 (**C**) or anti-cyclin D1 antibody to probe USP13 (**D**). **E** GST pull-down assay was used to detect the interaction between USP13 and cyclin D1 in vitro. **F** The colocalization of USP13 and cyclin D1 was detected with immunofluorescence analysis. Representative images were shown. **G** Schematic diagram of cyclin D1 and USP13 and their truncation mutants. **H**, **I** Coimmunoprecipitation analysis of the interaction between cyclin D1 and USP13 truncation mutants. The experimental procedures were the same as those in **A** and **B**.

To gain insights into USP13-mediated regulation of cyclin D1, we checked whether USP13 interacted with cyclin D1. We transfected Myc-tagged cyclin D1 (Myc-cyclin D1), together with or without Flag-tagged USP13 (Flag-USP13) into HEK293T or different GC cells and carried out exogenous Co-IP assay. As shown in Fig. 5A, Myc-cyclin D1 could be detected in Flag-USP13 immunoprecipitates using an anti- FLAG antibody in the cells transfected with Myc-cyclin D1 and Flag-USP13. Consistently, Flag-USP13 could be pulled down by Myc-cyclin D1 with an anti-Myc antibody (Fig. 5B). We then tested the endogenous interaction and found that cyclin D1, but not cyclin D2 and cyclin D3, could be pulled down by USP13 antibody (Fig. 5C and Supplementary Fig. S5A) and USP13 could also be pulled down by cyclin D1 antibody (Fig. 5D). To detect the direct interaction between USP13 and cyclin D1, we performed GST-pulldown assays with the recombinant GST-USP13 protein. The results showed that cyclin D1 can be pulled down by purified GST-USP13 protein (Fig. 5E). Moreover, the results of immunofluorescence assays indicated that USP13 could colocalize with cyclin D1 in GC cells (Fig. 5F).

To determine which region of cyclin D1 is critical for interacting with USP13, we constructed two cyclin D1 deletion mutants (Fig. 5G) and performed Co-IP experiments. The results indicated that the N-terminal but not the C-terminal of cyclin D1 could interact with USP13 (Fig. 5H). USP13 is composed of an UBP (ubiquitin-specific processing protease) -type zinc finger, an USP and two UBA (ubiquitin-associated/translation elongation factor EF1B, aminoterminal) domains [20]. We constructed five USP13 truncated mutants (Fig. 5G) and transfected Myc-cyclin D1, together with different Flag-tagged USP13 mutants into HEK293T cells. As shown in Fig. 5I, deletion of the USP13 UBA domain ablated the interaction between USP13 and cyclin D1 and the mutant containing UBA domain, but not USP and UBP domain, could interact with Myc-cyclin D1,indicating the UBA domain is crucial for interacting with cyclin D1. Unexpectedly, compared with the full-length USP13, deletion of the UBP domain obviously decreased the interation with cyclin D1, indicating that the UBP domain might play some positive role in the interaction between USP13 and cyclin D1.

### USP13 removed K48-linked polyubiquitination chain of Cyclin D1

**Fig. 6:**
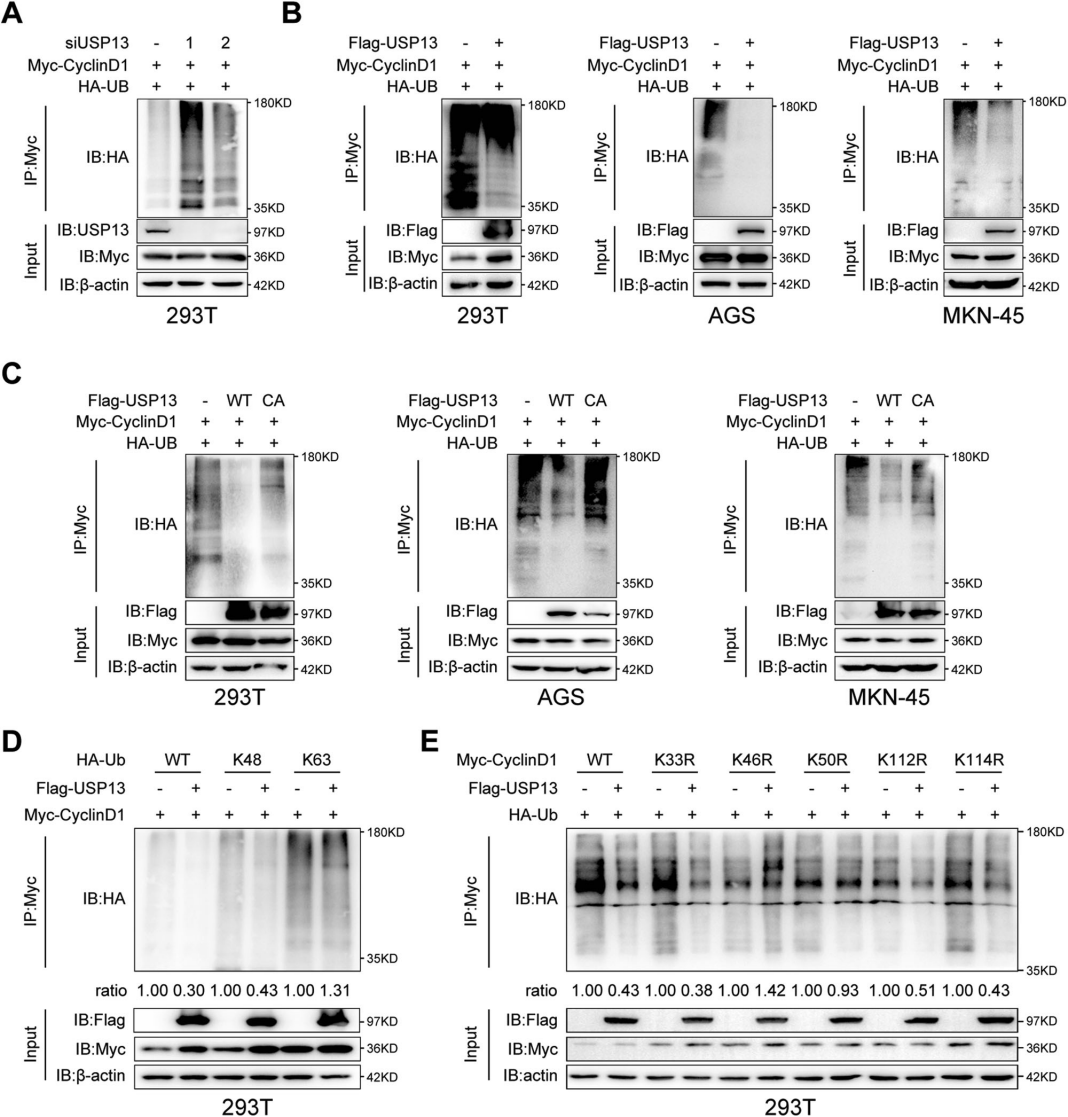
USP13 deubiquitinates cyclin D1. **A** The ubiquitination of cyclin D1 was analyzed using immunoprecipitation assay in HEK293T cells transfected with Myc-cyclin D1 and HA-Ub in the presence of USP13 siRNA or not. The ubiquitination of cyclin D1 was analyzed using immunoprecipitation assay in HEK293T cells and GC cells transfected with Myc-cyclin D1 and HA-Ub in the presence of WT Flag-USP13 (**B**, **C**) or Flag-USP13 mutant C345A (**C**). **D** Immunoprecipitation assay was used to analyze the ubiquitination of cyclin D1 in HEK293T cells transfected with Myc-cyclin D1 and HA-Ub or its lysine residue mutants HA-Ub K48 or HA-Ub K63 together with or without Flag-USP13. **E** Immunoprecipitation assay was used to analyze the ubiquitination of cyclin D1 in HEK293T cells transfected with HA-Ub and Myc-cyclin D1 or its lysine residue mutants (K33R, K46R, K50R, K112R, K114R) in which the lysine at K33, K46, K50, K112, K114 was replaced with arginine in the presence of Flag-USP13 or not.

To further define whether USP13 deubiquitinated cyclin D1, we transfected USP13 siRNAs or negative control siRNA together with Myc-cyclin D1 and HA-Ub into HEK293T cells and performed Co-IP assays. As shown in Fig. 6A, knockdown of USP13 with siRNA increased the ubiquitination level of cyclin D1 in HEK293T cells. In contrast, overexpression of Flag-USP13 decreased the ubiquitination level of cyclin D1 in HEK293T cells and GC cells (Fig. 6B). To further investigate whether the DUB activity of USP13 is essential for the effect, we transfected WT-USP13 or USP13-C345A mutant together with Myc-cyclin D1 and HA-Ub into HEK293T cells and GC cells. Co-IP assays results showed that the mutant USP13-C345A failed to decrease cyclin D1 ubiquitination level (Fig. 6C), indicating that the DUB activity of USP13 is indispensable for the deubiquitination of cyclin D1. To ascertain the deubiquitination types of cyclin D1 by USP13, we co-transfected Myc-cyclin D1 and HA-Ub K48 or K63 mutants, in which all lysine residues except the one at position 48 or 63 are replaced with arginine, together with or without Flag-USP13 into HEK293T cells. As shown in Fig. 6D, USP13 decreased the ubiquitination of cyclin D1 in the presence of WT and the K48-Ub mutant but not the K63-Ub mutant, indicating that the USP13 mainly removes the K48-linked polyubiquitination chains on cyclin D1.

Given that USP13 interacted with the N-terminal domain of cyclin D1, we conjectured that USP13 may remove the polyubiquitination chains linked to lysine (K) residues in the N-terminal domain of cyclin D1. There are 12 lysine residues in the N-terminal cyclin D1 (aa 1–153). Among these lysine residues, five distinct lysines (K33, K46, K50, K112 and K114) were detected as potential sites of ubiquitylation according to previous report [23]. We mutated each lysine residue from the five sites into arginine (R). We transfected Myc-tagged WT-cyclinD1 or different mutants into HEK293T cells together with HA-Ub and Flag-USP13. As shown in Fig. 6E, the mutant K46R and K50R abrogated USP13-mediated deubiquitination, indicating that USP13 mainly removed the polyubiquitination chain of cyclin D1 at K46 and K50.

### USP13 knockdown impaired the tumorigenesis of GC cells in vivo

**Fig. 7:**
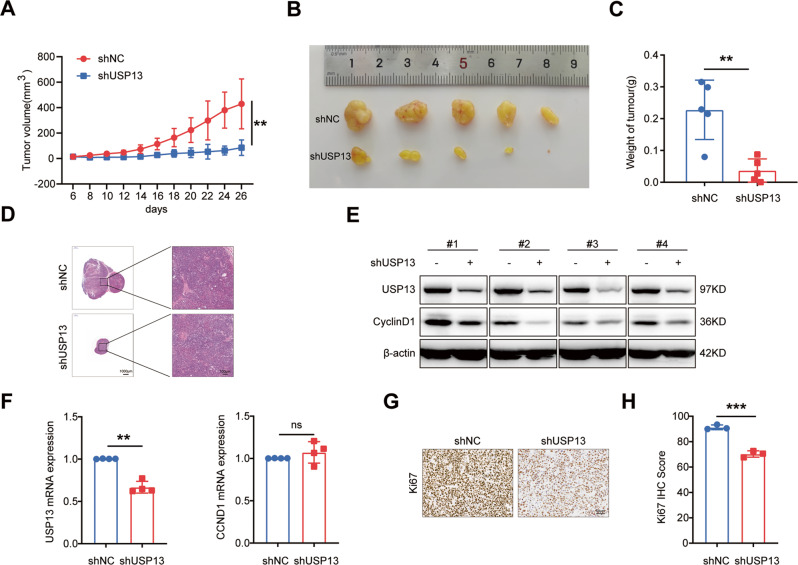
USP13 knockdown suppresses the tumor growth in vivo. **A** Tumor growth curves were plotted in the nude mice subcutaneously injected with USP13-depleted GC cells (shUSP13) or the negative control cells (shNC). **B** The tumors dissected from the nude mice were photographed and shown. A ruler was used to indicate the size of the tumors. **C** Statistical analysis of the tumor weight. The data are the means ± SD from 5 mice. **D** Hematoxylin and eosin (HE) staining was used to detect the tumors. **E** Western blot analysis of the expression of USP13 and cyclin D1 in the tumors. **F** qRT-PCR analysis of the expression of USP13 and cyclin D1 in the tumors. **G** IHC analysis of Ki-67 expression in the tumors. **H** Quantification and statistical analysis of the Ki-67 IHC scores. The data are the means ± SD. **p < 0.01; ***p < 0.001.

To investigate whether USP13 knockdown suppressed the tumorigenesis of GC cells in vivo, we injected MKN-45 GC cells which were stably knocked-down USP13 (shUSP13) or the negative control (shNC) into the subcutaneous of the nude mice. The results indicated that the tumor volume and tumor weigh in shUSP13 group were significantly smaller and lighter than those in the shNC group (Fig. 7A–C). HE staining validated that all tumors were solid tumors (Fig. 7D). Western blot results indicated that compared with the control group, the protein expression of USP13 and cyclin D1 decreased in the tumors of the shUSP13 group (Fig. 7E), whereas the mRNA level of cyclin D1 had no obvious changes (Fig. 7F). Immunohistochemistry (IHC) analysis showed that the expression of nucleus related antigen (Ki67) was significantly decreased in the shUSP13 group (Fig. 7G, H).

### USP13 regulated GC cell cycle progression and cell proliferation via cyclin D1

**Fig. 8:**
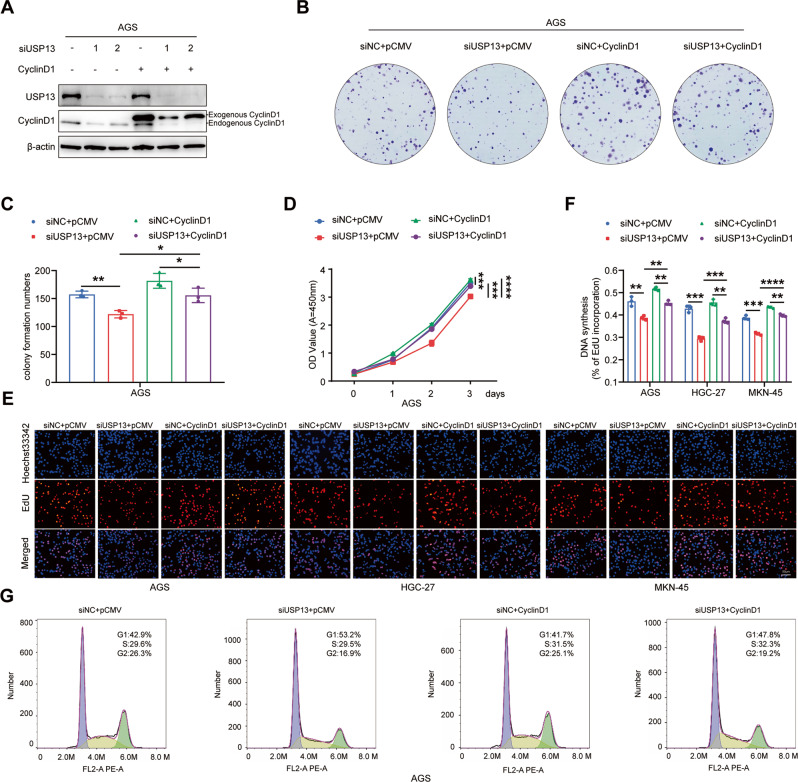
Cyclin D1 is involved in USP13-mediated biological function. **A** Western blot was used to analyze the protein expression levels of USP13 and cyclin D1 in different transfected GC cells. **B** Detection of the colony formation ability in GC cells with different transfections, Representative results were shown. **C** Statistical analysis of the colony formation number in transfected GC cells. The data are the means ± SD from 3 independent experiments. **D** CCK-8 assay was used to detect cell proliferation ability. The data are the means ± SD from 3 independent experiments. **E** EdU assay was used to determine the cell proliferation ability in transfected GC cells. The representative results were showed. **F** Statistical analysis of the EdU-positive cell ratio in GC cells. The data are expressed as the means ± SD from three independent experiments. **G** Flow cytometry was used to detect the cell cycle distribution in GC cells with different transfections. The representative results were showed. *p < 0.05; **p < 0.01; ***p < 0.001; ****p < 0.0001.

To further investigate whether cyclin D1 was involved in USP13-mediated biological roles in GC cells, we carried out rescue experiments. We cotransfected USP13 siRNA and cyclin D1 expression vector into GC cells (Fig. 8A) to determine whether the biological roles mediated by USP13 siRNA could be blocked by cyclin D1. Colony formation, CCK-8 and EdU experimental results showed that cyclin D1 could partially abrogated USP13 siRNA-mediated inhibition of colony formation ability (Fig. 8B, C) and cell proliferation ability (Fig. 8D–F). Flow cytometry results indicated that cyclin D1 could partially reversed the cell cycle arrest in G1 phase mediated by USP13 siRNA (Fig. 8G).

## Discussion

GC remains one of the most lethal cancers in the world [1]. Exploring the regulatory mechanism of the tumor and providing promising therapeutic target for GC patients is urgent and necessary. Cyclin D1 plays a critical role in the regulation of cell cycle G1/S phase progression and has been regarded as an important proto-oncogene in a large number of studies [23–26]. The overexpression or amplification of cyclin D1 contributes to uncontrolled cellular proliferation and malignant transformation. Dysregulation in the ubiquitin-dependent proteasome degradation is the important reason for the increased levels of cyclin D1 in tumors. Several ubiquitin ligases [27–31] (such as, FBX4, FBXO31, β-TrCP, FBXW8) and deubiquitinases [23, 32–34] (USP22, USP2, USP5, USP10) have been identified to regulate cyclin D1 degradation in different cancer cells. These epigenetic enzymes have great potential for development of targeted drugs. In the present study, we identified USP13 as a novel deubiquitinase for cyclin D1 in GC cells.

USP13 is extensively involved in various cellular processes, including the occurrence and progression of tumor. USP13 may function as a tumor suppressor or an oncogene in different tumor types [16–22]. In this study, we investigated the expression and biological roles of USP13 in GC. We used TCGA database to analyze the expression in different tumor types and found that USP 13 is up-regulated in most of the digestive system tumors, including GC. Furthermore, the expression of USP13 is positively related to tumor grades of GC. Our clinical data confirmed that the protein level of USP13 is increased in human GC tissues compared with the adjacent noncancerous tissues. Kaplan–Meier Plotter analysis indicated that the higher expression of USP13 is associated with shorter overall survival in GC patients. We further found that suppression of USP13 resulted in the cell cycle arrest in G1/S phase and the inhibition of the cell proliferation and colony formation ability in vitro. In contrast, overexpression of wild-type USP13, but not the enzymatically inactive mutant C345A promoted the cell cycle progression, cell proliferation and colony formation ability, indicating the DUB activity of USP13 is essential for the biological functions. Moreover, in vivo experiments in nude mouse also verified the tumor- promoting activity of USP13. To the best of our knowledge, this is the first study to elucidate the function of USP13 in cell cycle progression and cell proliferation in GC. These results provide evidence to support that USP13 serves as an oncogene in GC. However, it is still unclear how USP13 is up-regulated in GC. It has reported that lipopolysaccharides (LPS) reduces USP13 stability via ubiquitin-proteasome degradation pathway in Kupffer cells [35]. Since *Helicobacter pylori* infection is regarded as the strongest risk factor for GC [36], it remains to be further explored whether USP13 can be affected by the LPS or other virulence factors produced by *Helicobacter pylori*.

It has been reported that USP13 regulates several target substrates, such as MYC [8], Aurora B [15], PTEN [17], MCL1 [19, 20], MITF [21], ZHX2 [22]. However, the role of USP13 in modulating cell cycle proteins has not been reported yet. In this study, we reported for the first time that USP13 stabilized and deubiquitinated cell cycle protein cyclin D1 in GC. First, we found that the expression of USP13 was closely associated with cyclin D1. Enforced expression of USP13 increased cyclin D1 protein level, while knockdown of USP13 reduced cyclin D1 protein level. But USP13 did not alter the mRNA level of cyclin D1. Besides, USP13 protein was positively associated with cyclin D1 protein abundance in human GC tissue samples, indicating that USP13-mediated cyclin D1 regulation was clinically relevant. Second, USP13 maintained the stability of cyclin D1 by inhibiting its proteasome degradation. Third, Co-IP, GST pull down and immunofluorescence assays validated the physical interaction between USP13 and cyclin D1. Fourth, USP13 removed the polyubiquitination chains of cyclin D1 in DUB activity dependent manner. Finally, restoration of cyclin D1 upon USP13 depletion partially rescued the biological effects mediated by USP13 depletion, which suggested that cyclin D1 is one of the substrates of USP13 to promote GC cell proliferation. Taken together, these results indicate that USP13 is a direct DUB of cyclin D1 to contribute to GC cell cycle progression and cell proliferation. USP13 shared 80% sequence similarity with USP5 [37, 38]. The regulation of USP5 to cyclin D1 in lung cancer cells has been reported by Zhang et al. [33]. But we didn’t find the regulatory effect of USP5 on cyclin D1 in GC cells (Fig.S6), indicating that cyclin D1 may be regulated by different DUBs in different tumor types. These results revealed that the regulation of DUBs to target substrates has cellular specificity. The report from Zhang et al. revealed that USP13 promotes the epithelial-mesenchymal transition (EMT) and metastasis in GC by deubiquitinating and stabilizing Snail in GC [10]. Thus, USP13 may regulate several substrates in GC, thereby contributing to the tumorigenesis and progression of the tumor.

In this study, we tried to map the regions that mediated the interaction between USP13 and cyclin D1. Our results showed that the N-terminal domain of cyclin D1 (aa1–153) and the UBA (aa 625–863) domain of USP13 mediated the interaction between USP13 and cyclin D1. Although the UBP domain of USP13 can’t directly bind with cyclin D1, deletion of the UBP domain obviously decreased the interaction with cyclin D1. We speculated that the UBP domain of USP13 may participate in maintaining the structural basis for the interaction between USP13 and cyclin D1. The report from Liu et al. [39] and Scortegagna et al. [40] revealed that the UBA domain is crucial for USP13 to bind the target substrates, gp78 [39] and Siah2 [40]. Sun et al. [37] reported that multiple domains of USP13 (either aa1–624 or aa625–863) but not the UBP domain (aa1–300) are the primary determinant for its association with STING. However, the result from Zhang et al. [20] showed that N-terminal of USP13 is essential for the physical interaction with MCL1. Therefore, both the N-terminal and the C-terminal of USP13 might have the potential to bind with target substrates. The bona fide association between USP13 and its target substrate may depend on their mutual recognition and structural matching.

In conclusion, we find that USP13 serves as a direct DUB for cyclin D1 to remove its K48-linked polyubiquitination chains. USP13 is highly expressed in GC tissues. The elevated expression of USP13 promotes the cell cycle progression and cell proliferation by maintaining the stability of cyclin D1. Our study identifies USP13 as a novel regulator of cyclin D1 and reveals a unique function of USP13 in the regulation of GC cell cycle and cell growth. Thus, USP13/cyclin D1 axis may be a promising target for therapeutic intervention against GC.

## Materials and methods

### Patient samples and ethical approval

With the consent of the patients, we collected 34 pairs of primary GC tissues and corresponding adjacent noncancerous tissues at Taian City Central Hospital in 2016–8. The specimens were collected after surgery and immediately frozen in liquid nitrogen and stored until use. All samples are for research purposes only. The detailed information of the patients was listed in Supplementary Table S1. The research was approved by the ethics committee of the School of Basic Medical Sciences, Shandong University (LL-201601020) and performed in accordance with the Declaration of Helsinki.

### Cell culture

The human GC cell lines AGS, HGC-27 were purchased from the Cell Resource Center, Institute of Biochemistry and Cell Biology at the Chinese Academy of Science (Shanghai, China), and MKN-45 cells from the National infrastructure of Cell Line Resource (Beijing, China). The cell features were previously described by Wang et al. [41]. Human embryonic kidney cell line HEK293T was generously provided by Professor Zhao Wei (Shandong University, Jinan, China). HGC-27, MKN-45 and HEK293T cells were cultured in RPMI-1640 medium and AGS cells in F12 medium. All of the cells were supplemented with 10% fetal bovine serum (FBS) and 100 U/mL penicillin-streptomycin (Life Technologies, USA) and cultured at 37 °C with 5% CO_2_.

### siRNAs and plasmids

The siRNAs specific targeting USP13 and the negative control siRNA were from Genepharma (Shanghai, China). The siRNA sequences are listed in Supplementary Table S2. The siRNA was transfected into cells using Lipofectamine 2000 (Invitrogen, USA).

The overexpression plasmid pRK5-FLAG-USP13 (Plasmid #61741) and pcDNA3-Myc-cyclin D1 (Plasmid #122300) were purchased from Addgene (Cambridge, USA). USP13 mutant with lost enzymatic activity (USP13-C345A), USP13 truncation mutants and cyclin D1 mutants were generated using 2× Phanta^®^ Flash Master Mix (Vazyme, Nanjing, China, cat. P520) with pRK5-FLAG-USP13 or pcDNA3-Myc-cyclin D1 (WT) as template. All primer sequences for the mutants are listed in Supplementary Table S3. All of the constructs were verified by sequencing. pTac-GST-USP13 (P39515) was purchased from MiaoLingPlasmid (Wuhan, China). HA-K48 Ubiquitin and HA-K63 Ubiquitin plasmids were generous gift from Professor Zhao Wei (Shandong University, Jinan, China). The plasmids were transfected into cells using Hieff Trans™ Liposomal Transfection Reagent (Yeasen, Shanghai, China). The experiment was performed according to the manufacturer’s instructions.

### RNA extraction, reverse transcription and qRT-PCR

Total RNA was isolated from GC cells using TRIzol reagent (Invitrogen, USA) according to the manufacturer’s protocol. cDNA was synthesized with random primers using a HiScript III 1st Strand cDNA Synthesis Kit (+gDNA wiper) (Vazyme, R312–02). qRT-PCR was performed using Bio-Rad CFX96TM Real-Time PCR System (Bio-Rad) and ChamQ SYBR qPCR Master Mix A. The primers were synthesized by BioSune (Shanghai, China). The primer sequences are listed in Supplementary Table S4. The relative RNA expression levels were analyzed using the 2-△△Ct method. β-actin was used as the internal control. All reactions were run in triplicate.

### Western Blot

Total proteins from cells were extracted with RIPA lysis buffer containing proteinase inhibitor and quantified with the BCA Protein Assay Kit (Beyotime, China). Then the proteins were subjected to SDS-PAGE and transferred to polyvinylidene difluoride (PVDF) membrane (Merck Millipore, Germany), which were blocked in 5% nonfat milk and then incubated with primary antibodies at 4 °C overnight. The membranes were washed in Tris-buffered saline with Tween, incubated with anti-mouse or anti-rabbit horseradish peroxidase-conjugated secondary antibody for 1 h at room temperature, and developed with the enhanced chemiluminescence method (Millipore, USA). β-actin (Proteintech, 66009–1-Ig, 1:7000) served as a loading control.

The primary antibodies against following protein were used in this study: USP13 (Proteintech, 16840–1-AP, 1:3000), cyclin D1 (EPITOMICS, 2261–1, 1:1000), cyclin D2 (Proteintech, 67048–1-Ig, 1:5000), cyclin D3 (Proteintech, 10845–1-AP, 1:2000), CDK2 (Abcam, ab32147, 1:10000), CDK4 (Proteintech, 11026–1-AP, 1:500), cyclin E (Santa Cruz, sc-377100, 1:1000), p21 (Proteintech, 103551-AP, 1:4000), p27 (Proteintech, 25614-AP, 1:4000). FLAG-tagged proteins were detected with anti-FLAG antibody (Sigma-Aldrich, F1804, 1:1000). Myc-tagged proteins were detected with anti-Myc antibody (Origene, TA150121, 1:1000), HA-tagged proteins were detected with anti-HA antibody (Cell Signaling Technology, 3724S, 1:1000). Full-length original western blots in this article are provided in Supplementary File.

### Co-immunoprecipitation (Co-IP) and GST Pull-Down assays

For exogenous Co-IP assay, HEK293T or GC cells were transfected with different expression vectors for 48 h. For endogenous IP assay, the cells were inoculated on 10 cm culture plates. The cells were lysed with IP lysis buffer containing 50 mM Tris-HCl (pH8.0), 150 mM NaCl, 10 mM KCl, 1.5 mM MgCl2, 0.5% NP-40, 10% glycerol, 1 mM EDTA and protease inhibitor and incubated with anti-Flag (Sigma-Aldrich, F1804, 1:400) or anti-Myc antibody (Origene, TA150121, 1:400) for exogenous Co-IP, and anti-USP13 or anti-cyclin D1 for endogenous IP, then followed by the addition of Protein A/G Magnetic Beads (MedChemExpress, HY-K0202) overnight at 4 °C on a vertical roller. The beads were washed five times with PBST buffer. Both lysates and immunoprecipitates were analyzed by immunoblotting.

In the GST pull-down assay, GST-USP13 was expressed in *Escherichia coli* BL21 (DE3) cells, which were induced by isopropyl-b-D-thiogalactoside (IPTG, MedChemExpress, HY-15921). Cyclin D1 expression vector was transfected into HEK293T cells. The GST tagged USP13 protein was purified by Glutathione Sepharose beads (Yeasen, 20562ES08) and then subjected to immunoprecipitation assay.

### MG132 or Cycloheximide (CHX) treatment

GC cells were transfected with plasmids or siRNAs for 48 h and then were treated with MG132 (10 μM, MedChemExpress, HY-13259) or CHX (100 μg/mL, Sigma-Aldrich) for different durations. The proteins in the cells were extracted with RIPA lysis buffer and subjected to SDS-PAGE followed by western blot.

### Immunofluorescence assay

GC cells transfected with pRK5-FLAG-USP13 and pcDNA3-Myc-cyclin D1 plasmids for 48 h were seeded on a glass coverslip. The cells were fixed for 25 min in 4% paraformaldehyde and permeabilized with 0.1% Triton X-100 (in PBS) for 10 min, followed by blocking with 5% BSA for 30 min at room temperature. Then the cells were incubated with primary antibodies against Flag (Sigma-Aldrich, 1:500) and Myc (ABclonal, AE070, 1:250) at 4 °C overnight. After three PBS washes, the cells were incubated with CoraLite594 (Proteintech, SA00013–3, 1:500) and CoraLite488 (Proteintech, SA00013–2, 1:500) conjugated secondary antibodies for 2 h at room temperature in the dark. Cells were washed three times with PBS in the dark, stained with DAPI and mounted in antifade mounting reagent (Beyotime, P0126). The immunofluorescent staining was observed with a confocal microscope (ZEISS Axio Scope. A1, Germany).

### Cell cycle analysis

The treated GC cells were cultured for 48 h and then used the flow cytometry to analyze the cell cycle distribution. Briefly, the cells were fixed with 70% pre-cooled ethanol for 24 h. After being washed with cold PBS, 2 ×10^6^ cells from each group were stained with propidium iodide (Solarbio, C0080) for 15 min. Then the stained cells were detected with flow cytometer. The cell cycle distribution was analyzed using the software FlowJo_v10.8.1.

### Cell proliferation and colony formation assays

Cell proliferation was detected with EdU and CCK-8 assays. The EdU assay was performed according to the protocol of the Cell-Light™ EdU Apollo®567 In Vitro Imaging Kit (RiboBio, Guangzhou, China). Briefly, the treated cells were seeded in 96-well plates and incubated with 50 μM EdU for 2 h at 37 °C. After being fixed with 4% paraformaldehyde, the cells were exposed to 100 μL of 1× Apollo® reaction cocktail and then incubated with 5 μg/mL Hoechst 33342 to stain cell nuclei. Images were captured using a fluorescence microscope (Olympus, Tokyo, Japan). The percentage of EdU-positive cells was defined as the proliferation rate. For the CCK-8 assay, the treated cells were seeded in 96-well plates and incubated with 100 μL 10% CCK-8 solution for 2 h at 37 °C. The absorbance was measured at 450 nm with an Infinite M200 spectrophotometer (Tecan).

For the colony formation assay, 500 GC cells with different treatment were seeded in a 6-well plate and incubated with RPMI-1640 or F12 medium containing 10% fetal bovine serum (FBS) at 37 °C with 5% CO_2_ for almost 10 days. Then the cells were washed with PBS, fixed in 4% paraformaldehyde for 30 min and stained with crystal violet for 30 min. All of the experiments were performed in triplicate.

### Lentivirus infection

The lentivirus harboring USP13 shRNA or negative control were purchased from Genechem (Shanghai, China). The GC cells were infected with the lentiviruses in the presence of polybrene. The infected cells were selected in medium containing 2 μg/mL puromycin (Sigma, USA).

### Xenograft tumor experiment

Female BALB/c nude mice (4–5 weeks old) were purchased from the Vital River Laboratory Animal Technology Company (Beijing, China). All mice were randomly assigned to two groups. MKN-45 cells (5 ×10^5^) with stable knockdown of USP13 or control were harvested and re-suspended in 100 μL PBS. Then the cells were subcutaneously injected into the mice. Tumor size was monitored by measuring the length (L) and width (W) of the tumor every 2 days with a caliper, and the tumor volume (V) was calculated with the formula V = 1/2 × L × W^2^. Twenty-six days after injection, the mice were euthanized, and the tumors were weighed. All mice experiments were approved by the ethics committee of the School of Basic Medical Sciences, Shandong University and performed in accordance with the guidance of animal experiments in the Laboratory Animal Center of Shandong University.

### Statistical analysis

The data were analyzed with GraphPad Prism v8.0 2 software using Student’s *t* test (two-tailed) or two-way ANOVA test. All experiments were repeated independently at least three times, with similar results obtained. *p* < 0.05 was considered statistically significant (ns: no significance, *p < 0.05, **p < 0.01, ***p < 0.001, ****p < 0.0001).

## Supplementary information

Supplemental Figures and Tables

Supplemental data for original WB results

## Data Availability

The datasets used in this study are available from the corresponding author on reasonable request.
