# Supplementary material for: USP13 deubiquitinates and stabilizes cyclin D1 to promote gastric cancer cell cycle progression and cell proliferation
Source: Oncogene. 2023 Jun 13;42(29):2249–62. doi: 10.1038/s41388-023-02739-x (PMC10348911; doi:10.1038/s41388-023-02739-x)
Supplement: Supplementary file 1 — Supplemental Figures and Tables [file 41388_2023_2739_MOESM1_ESM.docx]

**Supplemental Figures and Tables for**

**USP13 Deubiquitinates and Stabilizes Cyclin D1 to Promote Gastric Cancer Cell Cycle Progression and Cell Proliferation**

**
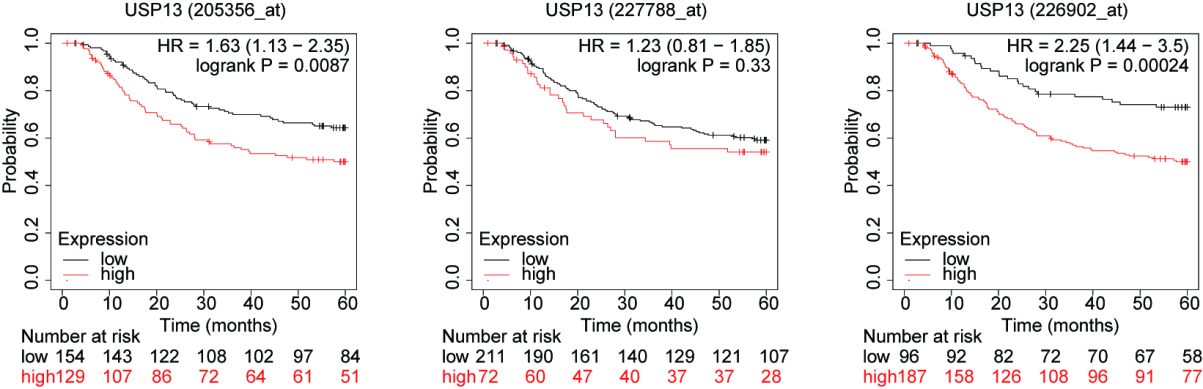
**

**Figure S1. Analysis of overall survival (OS) of GC patients from GSE62254 dataset in Kaplan–Meier Plotter database. The results are from three different Affymetrix probes.**

**
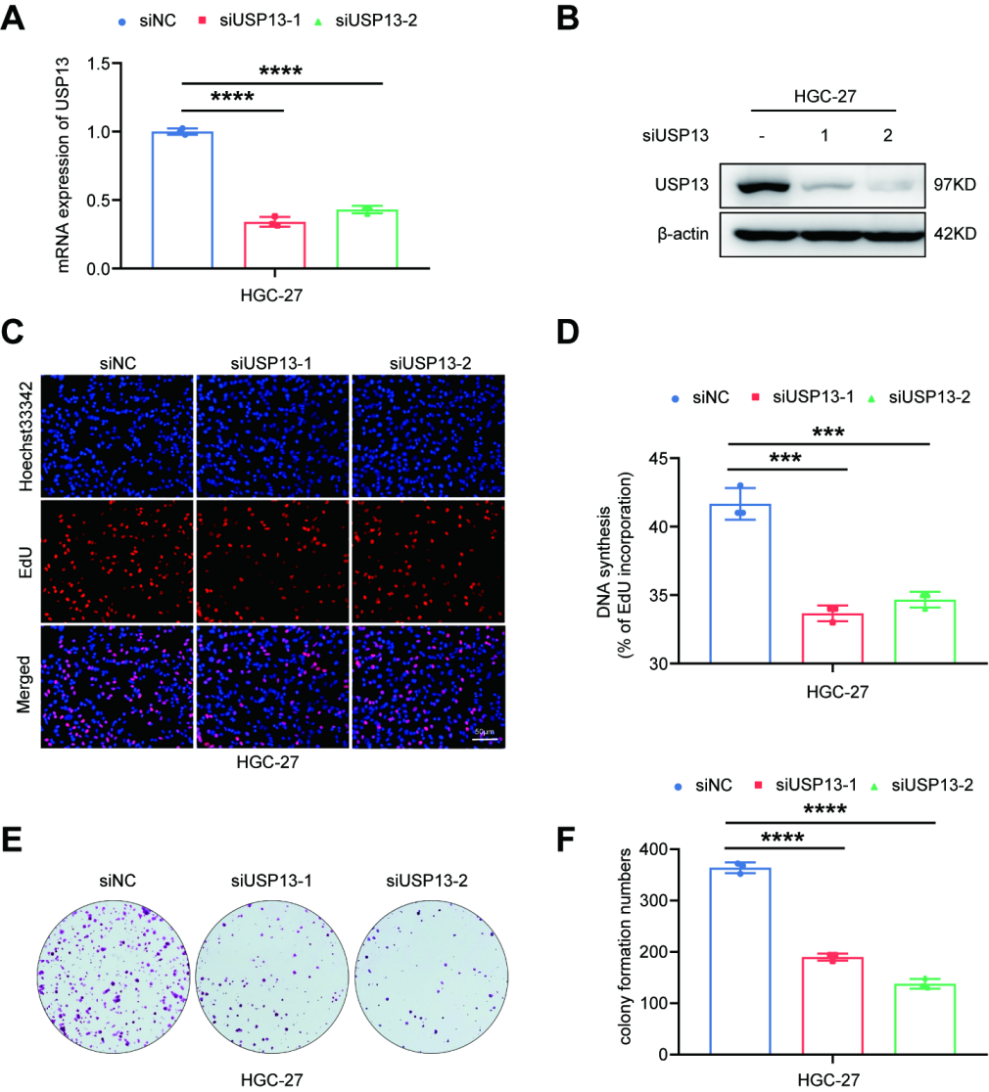
**

**Figure S 2. Knockdown of USP13 inhibits cell proliferation and colony formation ability in HGC-27 cells.**

A. qRT-PCR was used to determine the mRNA level of USP13 in HGC-27 cells transfected with negative control siRNA (siNC) or USP13 siRNA (siUSP13).

B. Western blot was used to detect the protein expression of USP13 in HGC-27 cells transfected with siNC or siUSP13.

C. EdU assay was used to determine the cell proliferation ability in transfected HGC-27 cells. The representative results were showed.

D. Statistical analysis of the EdU-positive cell ratio in GC cells. The data are expressed as the means ± SD from three independent experiments.

E. Colony formation ability was determined in transfected HGC-27 cells. Representative results were shown.

F. Statistical analysis of the colony formation number. The data are the means ± SD from three independent experiments. ****p* < 0.001, *****p* < 0.0001.

**
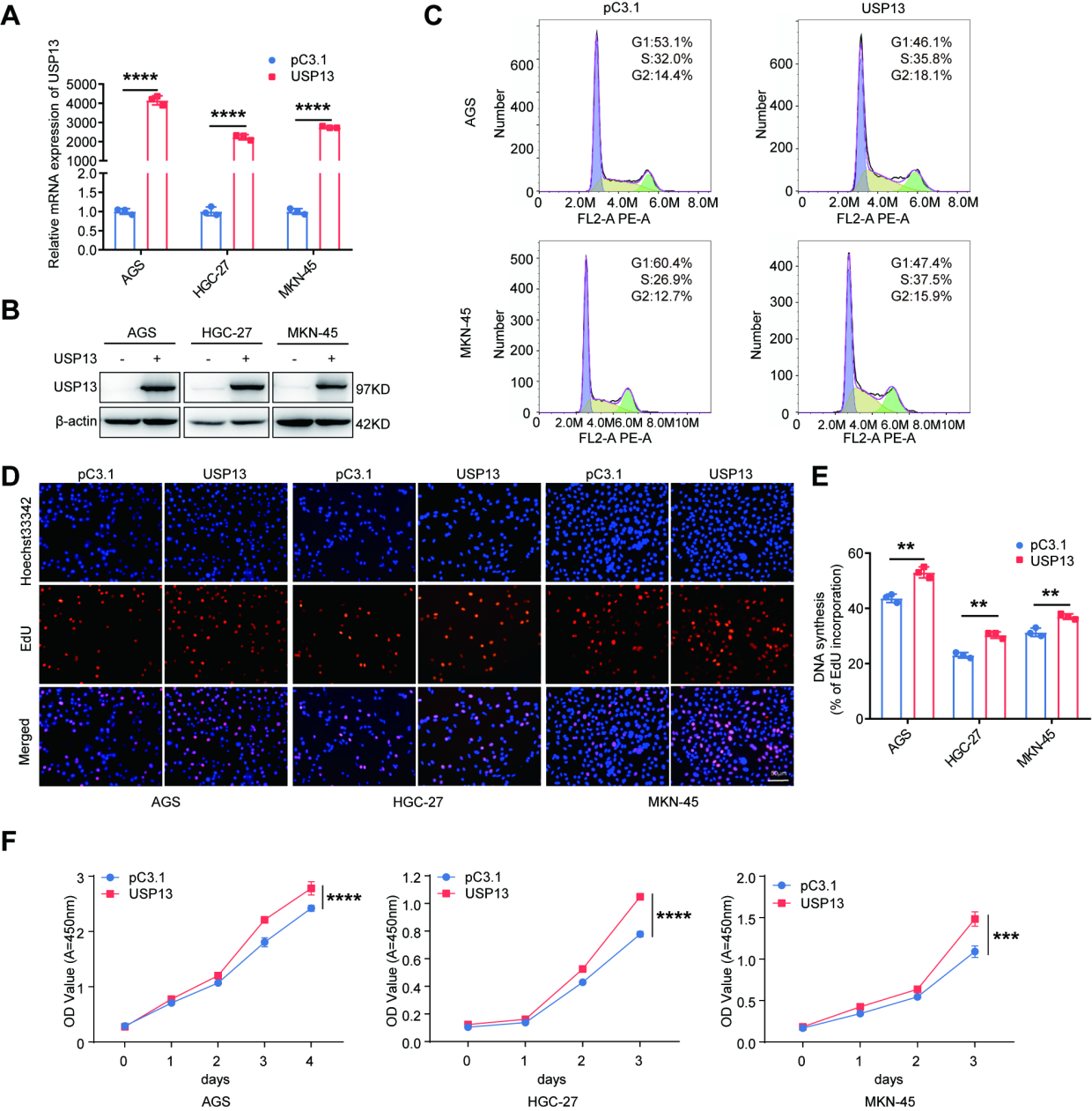
**

**Figure S 3. Overexpression of USP13 promotes cell cycle progression and cell proliferation ability in GC cells.**

A. qRT-PCR was used to determine the mRNA level of USP13 in GC cells transfected with USP13 overexpression (USP13) or control plasmid (pC3.1).

B. Western blot was used to analyze the expression of USP13 in GC cells transfected with USP13 overexpression plasmid or not.

C. Cell cycle distribution in GC cells with different transfection was determined using flow cytometry. Representative results were shown.

D. The cell proliferation ability in transfected GC cells was detected with EdU assay. The representative results were showed.

E. Statistical analysis of the EdU-positive cell ratio in GC cells. The data are the means ± SD from three independent experiments.

F. Cell proliferation ability was determined with CCK-8 assay in different transfected GC cells. The data are the means ± SD from three independent experiments. ***p* < 0.01, ****p* < 0.001,*****p* < 0.0001.

**
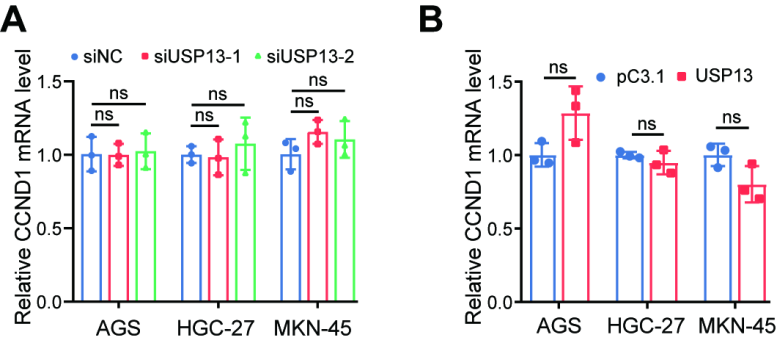
**

**Figure S 4. Effect of USP13 knockdown or overexpression on the mRNA level of cyclin D1 in GC cells.**

A. qRT-PCR was used to determine the mRNA level of cyclin D1 in GC cells transfected with negative control siRNA (siNC) or USP13 siRNA (siUSP13).

B. qRT-PCR was used to determine the mRNA level of cyclin D1 in GC cells transfected with USP13 overexpression vector (USP13) or empty vector (pC3.1). ns:no significance.


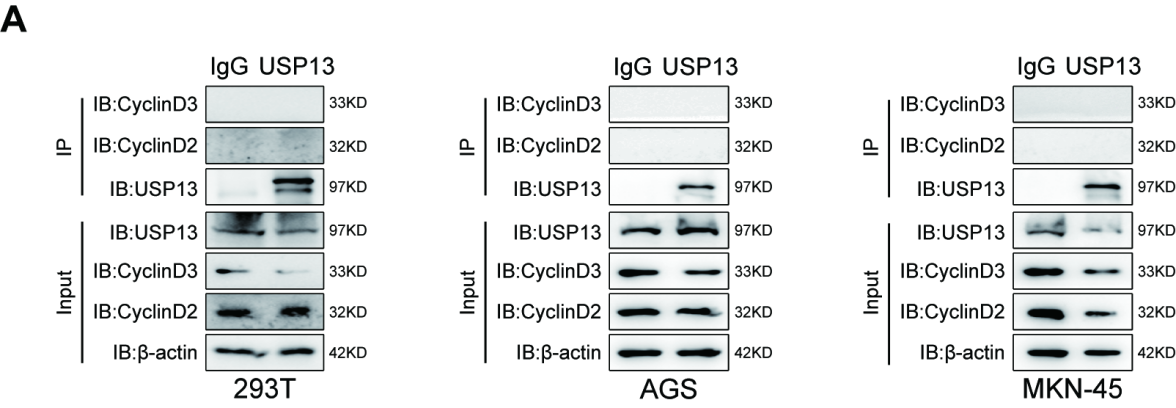


**Figure S 5. The endogenous interaction between USP13 and cyclin D2 or cyclinD3 in HEK293T and GC cells.**

1. IP assay was used to detect the endogenous interaction between USP13 and cyclin D2 or cyclinD3 in HEK293T cells and GC cells using anti-USP13 antibody.


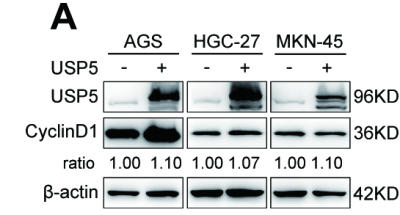


**Figure S6.The regulatory effect USP5 on cyclin D1 in GC cells.**

A. Western blot was used to detect the protein levels in GC cells transfected with USP5 expression vector.

**Table S1. Clinicopathologic characteristics of GC patients**

| **No.** | **Gender** | **Age（yr）** | **TNM** | **Size（diameter, cm）** |
| --- | --- | --- | --- | --- |
| 1 | F | 52 | T4aN3bM1 | 4.5 |
| 2 | F | 59 | T3N3bM1 | 5.5 |
| 3 | M | 55 | T4aN3bM0 | 7.5 |
| 4 | M | 45 | T4aN3aM1 | 10 |
| 5 | F | 66 | T4aN3aM0 | 3 |
| 6 | M | 73 | T4aN3bM0 | 7 |
| 7 | M | 57 | T1bN0M0 | 1 |
| 8 | M | 65 | T3N0M0 | 7 |
| 9 | F | 60 | T3N3aM0 | 2.5 |
| 10 | M | 62 | T4N2M0 | 6 |
| 11 | F | 46 | T4aN1M0 | 7 |
| 12 | M | 58 | T4aN1M0 | 2 |
| 13 | F | 69 | T3N1M0 | 3 |
| 14 | M | 51 | T3N2M0 | 3 |
| 15 | M | 75 | T3N3aM0 | 6 |
| 16 | F | 49 | T4aN1M0 | 3 |
| 17 | F | 77 | T2N0M0 | 1 |
| 18 | M | 52 | T2N1M0 | 2 |
| 19 | F | 40 | T3N3bM0 | 6 |
| 20 | F | 30 | T3N0M0 | 2.2 |
| 21 | M | 80 | T4aN1M0 | 8 |
| 22 | F | 56 | T1bN0M0 | 3 |
| 23 | F | 57 | T3N3aM0 | 5.5 |
| 24 | M | 64 | T3N2M1 | 5 |
| 25 | M | 74 | T2N1M0 | 2 |
| 26 | M | 66 | T4N3M0 | 9 |
| 27 | F | 69 | T3N3aM0 | 4 |
| 28 | F | 56 | T2N0M0 | 6 |
| 29 | M | 54 | T4aN3aM0 | 7 |
| 30 | F | 65 | T4aN3aM0 | 12 |
| 31 | M | 52 | T4aN2M0 | 10.5 |
| 32 | F | 66 | T3N1M0 | 8 |
| 33 | M | 59 | T4N3M0 | 6.5 |
| 34 | F | 37 | T4aN3aM1 | 13 |

**Table S2.siRNA sequences used in this study**

| **Name** | **siRNA Sequence** |
| --- | --- |
| USP13 siRNA-1 | 5’-GAAGAUGGGUGAUUUACAA-3’ |
| USP13 siRNA-2 | 5’-GCACUGGAUUGGAUCUUUA-3’ |
| Negative control | 5’-CCUACAUCCCGAUCGAUGAUGUUGA-3’ |

**Table S3. Primer sequences for mutant construction in this study**

| **Name** | **Primer Sequence** |
| --- | --- |
| USP13-C345A | F:5’- GCAACAGCGCCTATCTCAGCTCTGTCATGCAGGC -3’ |
|  | R:5’- TGAGATAGGCGCTGTTGCCCAGGTTCTTCAGAC -3’ |
| USP13-△UBA | F:5’- GGAGAGGAAGCGGCCGCTCGAGCATGCATCTAG -3’  R:5’- GGCCGCTTCCTCTCCTGGCTGTAACCCCCTGGC -3’ |
| USP13-UBA | F:5’- GGGTCGACAGAACTTCCAGACATCAGCCCCCCC -3’ |
|  | R:5’- AAGGACGACGATGACAAGGGGTCGACAGAACTT -3’ |
| USP13-△UBP | F:5’- GGGTCGACAACAGAGAATGGGCTCCAGGACAAT-3’ |
|  | R:5’- CTCTGTTGTCGACCCCTTGTCATCGTCGTCCTT -3’’ |
| USP13-UBP | F:5’- ATGCATGGGGCGGCCGCTCGAGCATGCATCTAG -3’ |
|  | R:5’- GGCCGCCCCATGCATATGAAGCATATCAATTCC -3’ |
| Cyclin D1-N | F:5’- AACCTGGCCCCCGGACTGCCTCCGGGCCTGCCA -3’ |
|  | R:5’- TCCGGGGGCCAGGTTCCACTTGAGCTTGTTCAC -3’ |
| Cyclin D1-C | F:5’ GAAGATCTGATGACCCCGCACGATTTCATTGAA -3’ |
|  | R:5’- GGTCATCAGATCTTCTTCAGAAATAAGTTTTTG -3’ |
| Cyclin D1-K33R | F:5’- ATGCTGCGCGCGGAGGAGACCTGCGCGCCCTC -3’ |
|  | R:5’- TCCTCCGCGCGCAGCATGGCCCGCAGCACCCG -3’ |
| Cyclin D1-K46R | F:5’- TACTTCCGCTGTGTGCAGAAGGAGGTCCTGCC -3’  R:5’- TGCACACAGCGGAAGTAGGACACCGAGGGCGC -3’ |
| Cyclin D1-K50R | F:5’- AAATGTGTGCAGCGCGAGGTCCTGCCGTCCATG -3’  R:5’- TCGCGCTGCACACATTTGAAGTAGGACACCGA -3’ |
| Cyclin D1-K112R | F:5’- CTCTCGCATGAAGGAGACCATCCCCCTGACGG -3’ |
|  | R:5’- TCTCCTTCATGCGAGAGGCCACGAACATGCAAG -3’ |
| Cyclin D1-K114R | F:5’- TAAGATGCGCGAGACCATCCCCCTGACGGCCG -3’ |
|  | R:5’- TGGTCTCGCGCATCTTAGAGGCCACGAACATGC -3’ |

**Table S4 Primer sequences for qRT-PCR in this study**

| **Name** | **Primer Sequence** |
| --- | --- |
| USP13  (162bp) | F:5’- TCTCCTACGACTCTCCCAATTC- 3’ |
|  | R:5’- CAGACGCCCCTCTTACCTTCT- 3’ |
| CCND1  (211bp) | F:5’- GATGCCAACCTCCTCAACGA - 3’ |
|  | R:5’- GGAAGCGGTCCAGGTAGTTC- 3’ |
| β-actin  (177bp) | F:5’- GAAGTGTGACGTGGA CATCC - 3’ |
|  | R:5’- CCGATCCACACGGAGTACTT - 3’ |
