# Supplementary figures and images for: USP13 deubiquitinates and stabilizes cyclin D1 to promote gastric cancer cell cycle progression and cell proliferation
Source: Oncogene. 2023 Jun 13;42(29):2249–62. doi: 10.1038/s41388-023-02739-x (PMC10348911; doi:10.1038/s41388-023-02739-x)

Supplemental data for original Western Blot results


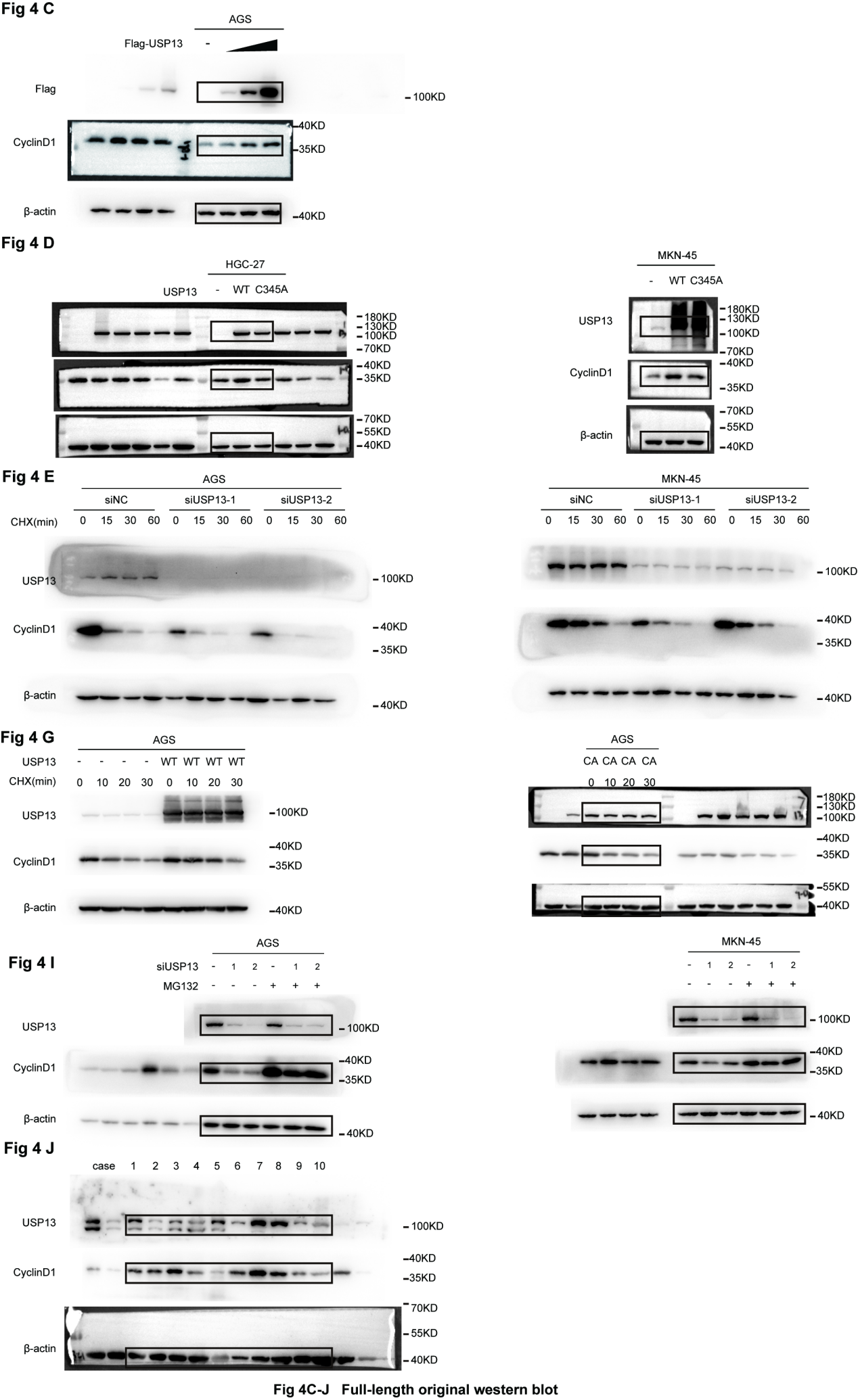


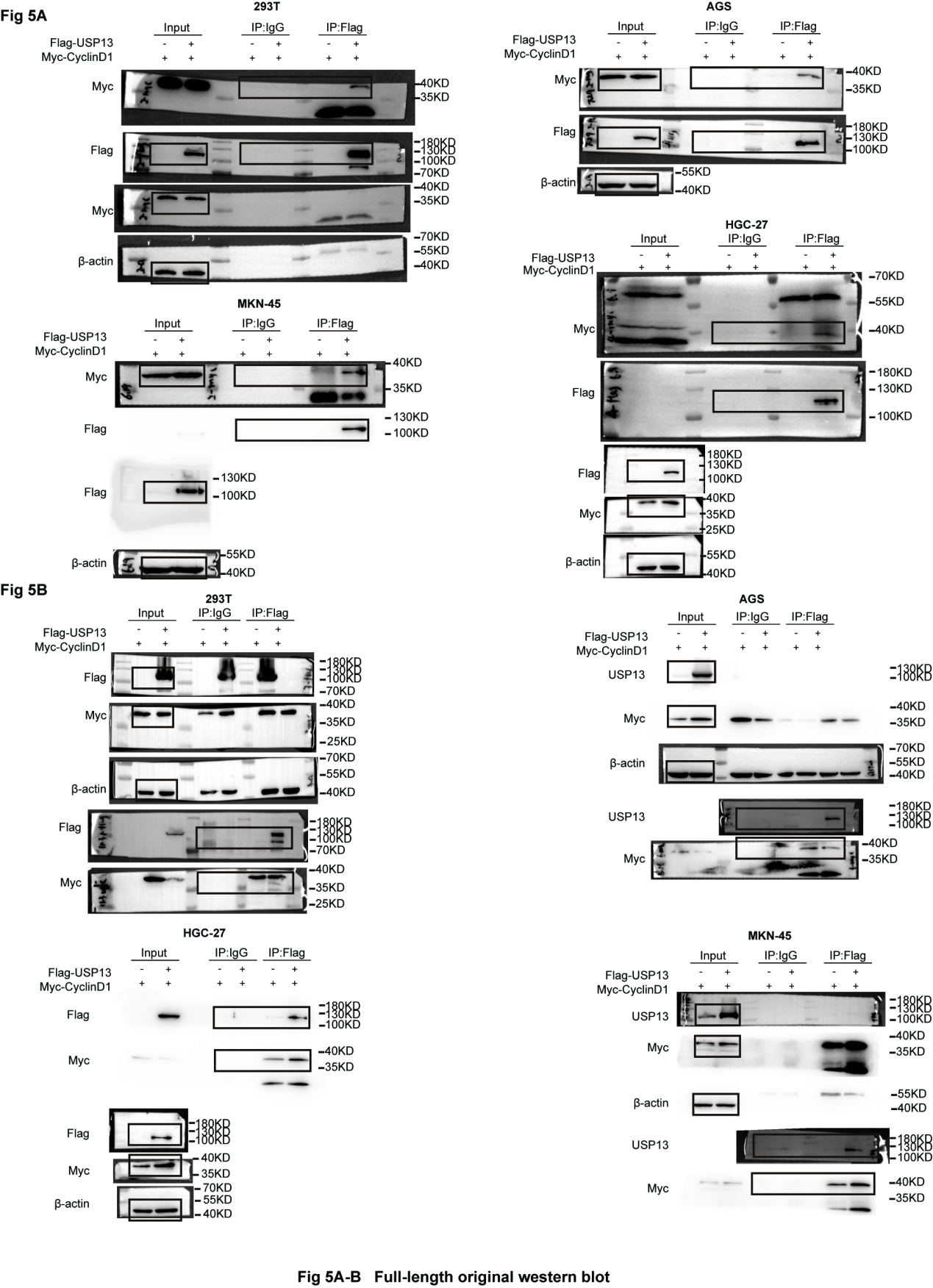


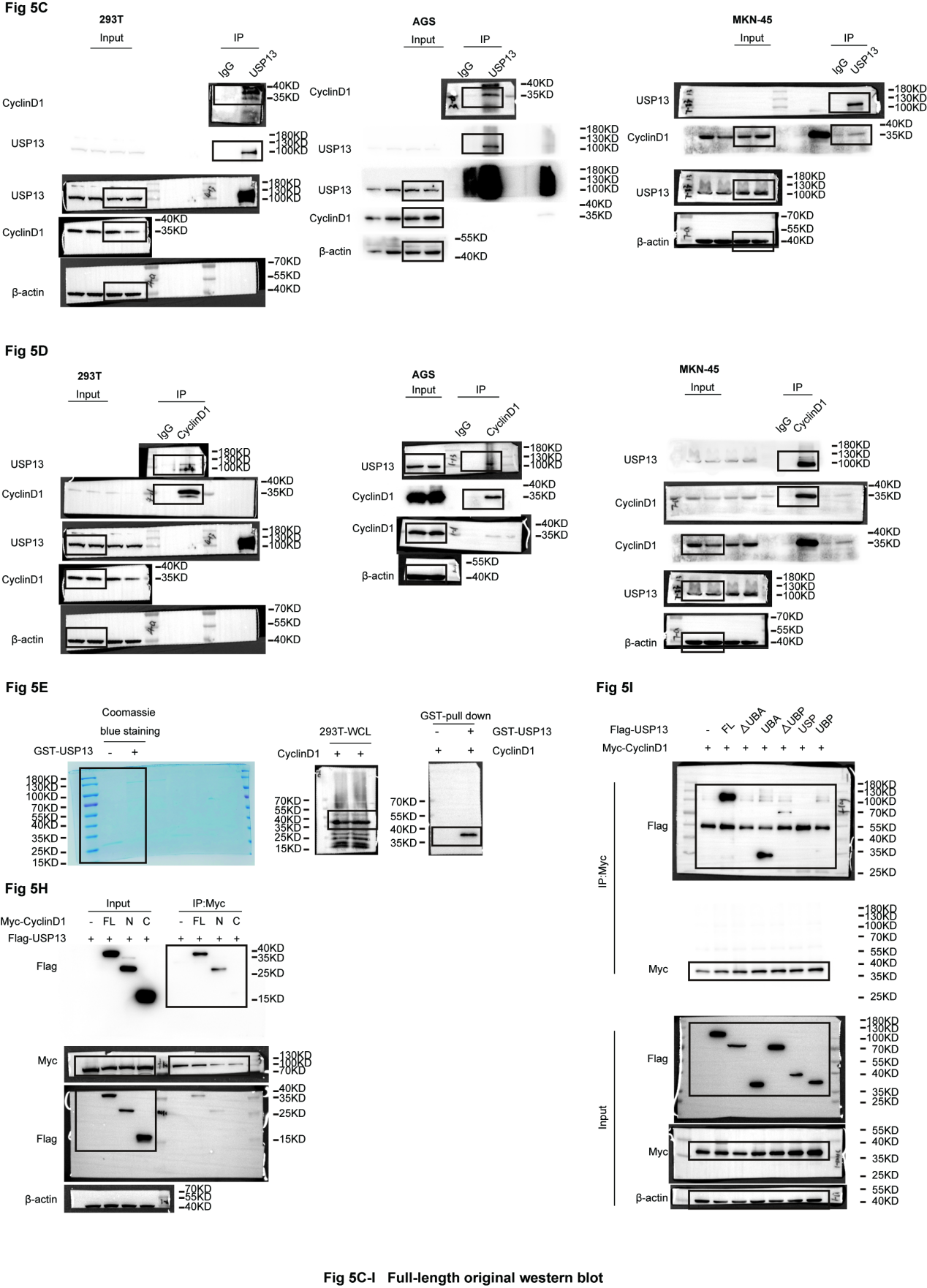


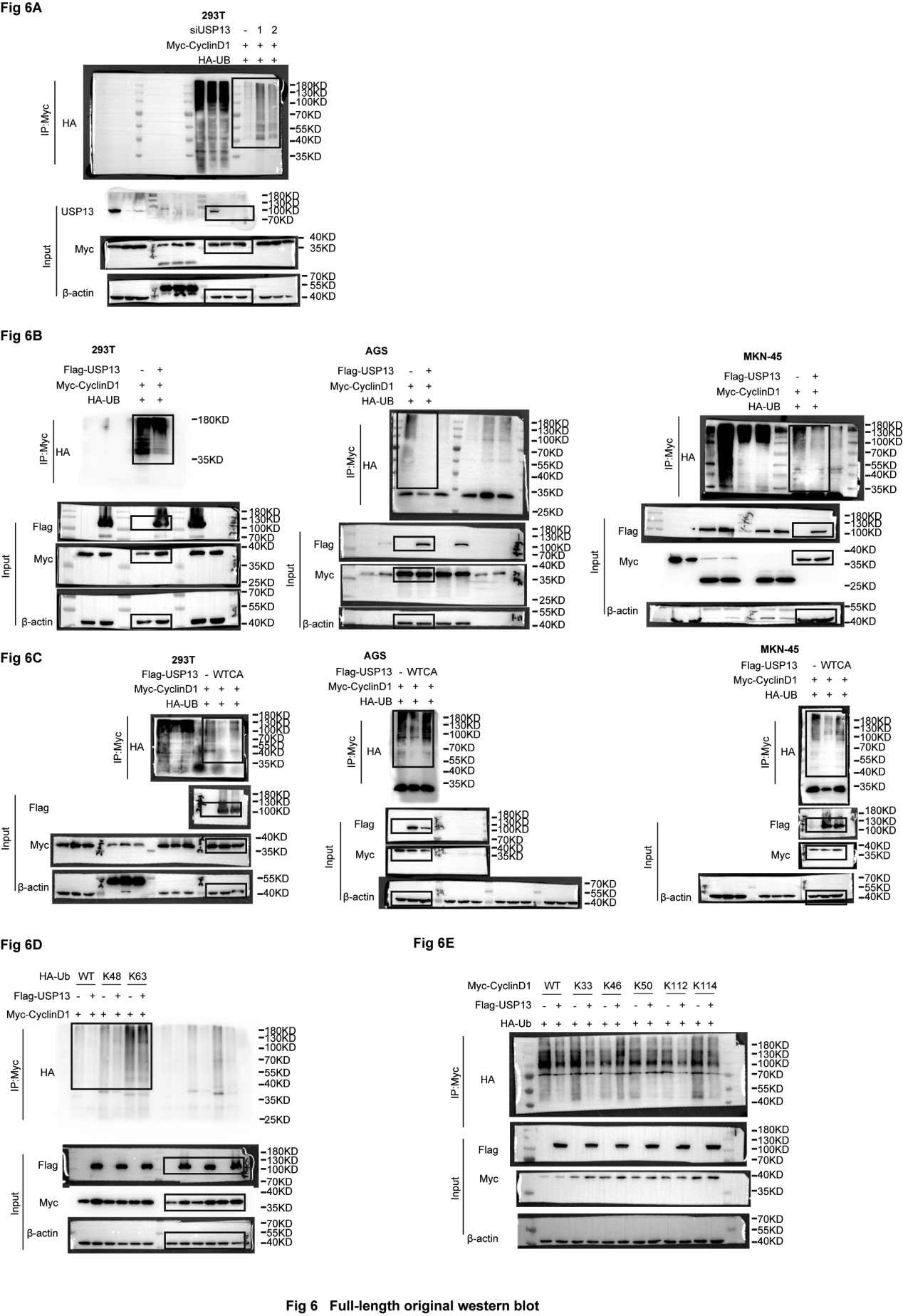


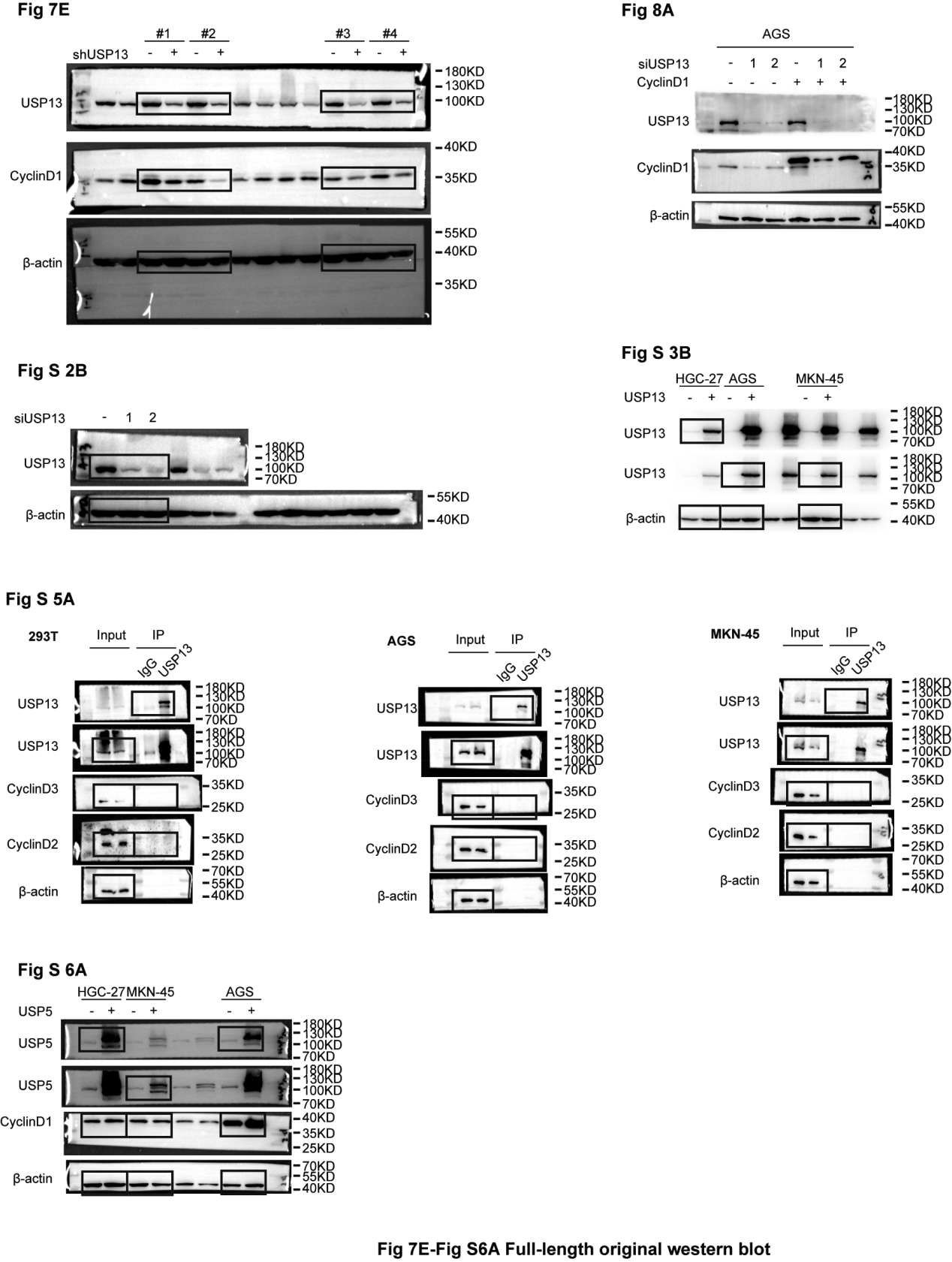

Supplement: Supplementary file 2 — Supplemental data for original WB results [file 41388_2023_2739_MOESM2_ESM.docx]
